# Validation of the Chinese version of the Reynolds’ suicidal ideation questionnaire: psychometric properties and its short version

**DOI:** 10.1186/1477-7525-12-33

**Published:** 2014-03-10

**Authors:** Yi Zhang, Paul Siu Fai Yip, King-wa Fu

**Affiliations:** 1 Department of Social Work and Social Administration, The University of Hong Kong, Hong Kong Special Administration Region, People’s Republic of China; 2 The Hong Kong Jockey Club Centre for Suicide Research and Prevention, The University of Hong Kong, Hong Kong Special Administration Region, People’s Republic of China; 3 Journalism and Media Studies Centre, The University of Hong Kong, Hong Kong Special Administration Region, People’s Republic of China

**Keywords:** Suicidal ideation questionnaire, Psychometric properties, Chinese society

## Abstract

**Background:**

This study aims to validate the Chinese version of the Reynolds’ Suicidal Ideation Questionnaire (SIQ) in a Chinese society and explore a convenient short version.

**Methods:**

A sample of 711 cases was derived from two territory-wide surveys of Hong Kong adolescents aged between 15 and 19 years old.

**Results:**

The SIQ and the Suicidal Ideation Questionnaire-Junior (SIQ-JR) demonstrated good reliability and concurrent validity among Hong Kong adolescents. However, the factor structure for both SIQ and SIQ-JR appeared to be unclear. A four-item short form of the SIQ-JR, namely, SIQ-JR-4, was proposed.

**Conclusion:**

The SIQ-JR-4 is an ideal substitute of the SIQ/SIQ-JR for future quick assessment of suicidal ideation in Chinese young adolescents.

## 

Suicide risk begins to increase since adolescent ages [1,2]. Early identification of suicidal adolescent is of great priority in suicide prevention, as suicidal ideation is a common antecedent of more severe outcomes like suicide attempts or completed suicide [3-6]. The efficacy of the screening for suicidal adolescents relies largely on the reliability, validity, and utility of the corresponding screening tool [4,7]. However, there is lack of a validated scale for evaluating severity of suicidal ideation among adolescents in Chinese-spoken societies, such as China, Tai Wan, and Hong Kong.

The Reynolds’ Suicidal Ideation Questionnaire (SIQ) is a 30-item self-administered measurement for assessing severity of suicidal ideation among adolescents [8]. The Suicidal Ideation Questionnaire Junior (SIQ-JR) is a 15-item short version extracted from the SIQ developed by the same author [8]. Both versions of the questionnaire have been commonly used in English-spoken societies with demonstrated good internal consistency, test-retest reliability, and construct validity [9-13]. Noticeably, the factor structure of both scales appeared to be unclear due to the inconsistent results of factor analysis [8,10,11,14]. In practical application, the lengthiness of the SIQ/SIQ-JR created excessive burden for respondents, affected the response rate, and undermined the efficacy of the screening. An even shorter version of the SIQ-JR is necessary for popularizing the application of this questionnaire.

In this study, the SIQ/SIQ-JR has been translated into Chinese in accordance with social and culture context. It aims to shed light on the psychometric characteristics of the Chinese version of the SIQ and SIQ-JR and also explore a short form for fast screening purpose. A community-based sample of adolescents in Hong Kong was employed in the analysis.

## Methods

### Participants

Data for the analysis of this study derived from two territory-wide surveys of Hong Kong residents. The sampling frame for both surveys was the Frame of Quarters, which is maintained by the Census and Statistics Department of Hong Kong. This is the most complete and up to date registry of residential addresses in the territory. Design of interviews and measurements used in both surveys were the same. Face-to-face interviews were carried out by trained interviewers. Sensitive questions like suicidal behaviors were self-reported by participants on a septe sheet of questionnaire.

Slightly different sampling procedures were employed for generating two samples. Sample 1 was an extracted group of 203 respondents aged 15-19 years from a survey of local residents aged 15-59 years conducted between December 2003 and July 2004. First, a household was randomly selected from the Frame of Quarters, and second, one household member was randomly selected to participate in the study. This survey yielded successful interviews with 2,219 respondents, giving a response rate of 62%. Gender and age distributions in this yielding sample were similar to that in the general population. Sample 2 was generated from another survey of local residents aged 15-19 years conducted in June-August 2004. A septe list of households from the Frame of Quarters was prepared and all adolescents aged between 15 and 19 years in these households were invited to be interviewed. A total of 511 adolescents participated in the study, yielding a response rate of 61% by household. Both samples in total yielded 714 respondents, among which, 3 had missing values in the SIQ. Hence, there were 711 valid cases for the analysis of this study. Written informed consent was obtained from all respondents for both surveys. Details of the two household surveys have been published elsewhere [7,15,16]. This study was approved by the Ethics Committee of the Faculty of Social Sciences, the University of Hong Kong.

### Measures

The surveys began with a face-to-face interview, in which all participants answered interviewer-administered questions about depression, hopelessness, and anxiety. They were then asked to finish a self-reported questionnaire that included questions on suicidal ideation and suicide attempt.

#### Suicidality

The self-reported booklet of questionnaire included a question “During the past 12 months, had you ever considered suicide?” and a question “During the past 12 months, had you ever attempted to commit suicide?” Participants with positive responses to these two questions were considered to have suicidal ideation and suicide attempt in the past 12 months, respectively.

Severity of suicidal ideation was assessed by the Chinese version of Suicidal Ideation Questionnaire (SIQ), indicated by the frequency of suicidal ideation within the past month [8]. The SIQ consists of 30 items. Each item is a 7-point scale, ranging from 0 (I never had this thought) to 6 (almost every day). Therefore, the total score of the SIQ ranges from 0 to 180. The SIQ includes content of a wide aspect of suicidal ideation, including specific wishes and plans of suicide (e.g., items like “I thought about killing myself” and “I thought about how I would kill myself”), the response and aspects of others (e.g., “I thought about how people would feel if I killed myself”), and morbid ideation (e.g., “I thought about people dying”). A shortened version of 15 items, the Suicidal Ideation Questionnaire-Junior (SIQ-JR), was developed for younger adolescents as a brief screening measure by the original author [8]. The Chinese version of the SIQ was forward and backward translated from English to Chinese by two bilingual clinical psychologists. The back translation had obtained the approval of W. M. Reynolds, the original author of the SIQ. Both the SIQ and SIQ-JR had been confirmed with good psychometric characteristics in samples from Western populations [8,9].

#### Depression

The severity of depressive symptoms was measured by the Chinese version of the Center for Epidemiologic Studies Depression (CES-D) Scale, a 20-item questionnaire [17,18]. Responses to each item indicate the frequency of depressive mood state. The CES-D is a 4-point scale that ranges from 0 (rarely or none of the time) to 3 (most or all of the time). Thus, the total score of the CES-D ranges from 0 to 60, with higher scores denoting a higher level of depression. Evidence supports the reliability and validity of this Chinese version of the CES-D [17,19,20].

#### Hopelessness

Hopelessness was assessed using the 20-item Chinese version of the Beck’s Hopelessness Scale (BHS) [21,22]. Unlike the original version of the BHS, its Chinese version expanded item responses from a Yes/No answer to a 6-point answer of “Strongly Agree, Moderately Agree, Slightly Agree, Slightly Disagree, Moderately Disagree, or Strongly Disagree” [21]. Hence, the total scores ranges from 20 to 120, with higher scores indicating higher level of hopelessness. The Chinese version of the BHS has been validated and widely used in Chinese-speaking populations [15,21,23].

#### Anxiety

The severity of anxiety symptoms was measured by a 7-item anxiety subscale of the Chinese Depression Anxiety Stress Scales (DASS) [24,25]. It is a 4-point self-report scale that measures anxiety symptoms during the past week. Summary score of the 7 items ranges from 7 to 28. Higher scores represent a higher level of anxiety mood states. The Chinese version of the Anxiety subscale of the DASS used in this study has been validated and used in Chinese-speaking populations [25-27].

### Statistical analysis

The Statistical Package for the Social Sciences (SPSS) for Windows, version 17.0 (SPSS Inc., Chicago, IL, USA), Analysis of Moment Structures (AMOS) 16.0 SEM software from SPSS [28,29], and R, version 2.15.0, developed by the R Core Team [30] were used for the statistical analyses. Since a skewed distribution of the SIQ and the SIQ-JR items was hypothesized, nonpmetric statistics were applied. Mann-Whitney U tests and Kruskal-Wallis analysis of variance by ranks were applied respectively to determine whether there were significant gender and age differences in the SIQ and the SIQ-JR median scores. Cronbach’s coefficient was deployed to measure internal consistency [31].

Factor analysis was performed to explore the factor structure for the SIQ and SIQ-JR. Although the design of the SIQ/SIQ-JR was lack of theoretically based factor structure, Reynolds [8], the originate author, had explored a three-factor structure for both versions of scales, through exploratory factor analysis (EFA). Confirmatory factor analysis (CFA) with AMOS was conducted for both scales to examine both one-factor and three-factor structures proposed by the original author. The results from the CFA were not satisfactory. EFA was thereafter performed to provide insight of the underlying factor structure of both scales as a reference for future studies in Chinese population samples. Varimax rotation method was occupied in the EFA.

A short version of the SIQ-JR was explored for fast screening. The items kept in the short version were expected to have high correlations with the original scale. Corrected item-total correlations were ranked and a cutoff of 0.80 yielded four items with highest correlations as a short version, namely SIQ-JR-4 [32,33]. Spearman’s rank correlation was used to examine the associations between the SIQ, SIQ-JR, and SIQ-JR-4 and related psychological assessments. Receiver operating characteristics (ROC) analyses were performed using the SIQ/SIQ-JR/SIQ-JR-4 scores to differentiate respondents with and without suicidal ideation and suicide attempts in the past 12 months septely. The performance of the SIQ, SIQ-JR, and SIQ-JR-4 in predicting suicidal ideation and suicide attempts was compared with that of depression, hopelessness, and anxiety. Discriminative power was indicated by the area under ROC curve (AUC) that an AUC closer to 1 represented a better discriminative power [34]. The equality of the AUCs between different measurements was tested using a nonpmetric method [35,36]. In addition, sensitivity and specificity analyses were deployed to assess the performance of measurements. The value that maximized the Youden index (defined as “sensitivity + specificity-1”) was set as the cutoff for each scale [37-40].

## Results

### Descriptive statistics

Out of 711 respondents, 44 reported suicidal ideation and 20 reported suicide attempts in the past 12 months. The prevalence rate (95% CI) was 6.2% (4.4-8.0%) and 2.8% (1.6-4.0%) respectively. The overall mean scores for the SIQ and SIQ-JR were 10.19 and 5.57 respectively. The distribution of both measurements’ scores was highly-skewed to the right (SIQ skewness = 4.25; SIQ-JR skewness = 3.84), which was consistent with previous findings [8]. This skewness was also suggested by the clear discrepancies (more than two folds differences) between overall median (SIQ median = 3; SIQ-JR median = 2) and mean scores (SIQ mean = 10.19; SIQ-JR mean = 5.57) for both scales. No significant age differences were found for the SIQ/SIQ-JR in both samples (Kruskal-Wallis Test, *P* > 0.05). However, significant gender differences were detected in both measurements: Female were more likely to report higher levels of suicidal ideation than male respondents (Mann-Whitney Test, *P* < 0.001).

### Reliability

The internal consistency of the SIQ/SIQ-JR was examined by employing Cronbach’s coefficient alpha, inter-item correlation, item-to-total scale correlation, and standard error of measurement (results not shown). Cronbach’s coefficients were found to be about 0.975 for the SIQ, and 0.951 for the SIQ-JR, in the overall, male, and female samples, which were similar in size to the findings reported by Reynolds [8]. The homogeneity of item content for the SIQ/SIQ-JR was supported by good item-total correlations, with all correlations higher than 0.5 and median item-total correlations higher than 0.7 in the overall, male, and female samples. There was very little variability in standard error values, since standard error of measurements were around 3 raw score points for the SIQ and 2 for the SIQ-JR. This was quite a small value in consideration of the large range of the SIQ (0-180) and the SIQ-JR (0-90), as well as the potential value interval on each item of 0-6.

### Factor analysis

Reynolds [8] proposed a three-factor structure for the SIQ: specific wishes and plans of suicide (including the SIQ items 1, 2, 3, 4, 7, 9, 10, 11, 12, 13, 14, 15, 16, 17, 18, 19, 20, 21, 22, and 23), the response and aspects of others (including the SIQ items 24, 25, 26, 27, 28, 29, and 30), and morbid ideation (including the SIQ items 5, 6, and 8). Reynolds also explored a three-factor structure for the SIQ-JR: minor suicidal ideation factor consistent with wishes that one were dead (including the SIQ-JR items 1, 11, 12, 13, 14, and 15), specific plans and desires for suicide (including the SIQ-JR items 2, 3, 4, 7, 8, 9, and 10), and morbid ideation (including the SIQ-JR items 5 and 6).

CFA was firstly carried out to test the goodness of fit of the one-factor and three-factor structures of the SIQ and the SIQ-JR respectively. Poor levels of goodness of fit were found in all models (chi-square-degree-of-freedom ratio: χ^2^/df > 10; root-mean-square error of approximation: RMSEA > 0.1; goodness-of-fit index: GFI < 0.9; and comptive fit index: CFI < 0.9). These results indicated that the proposed factorial structures of the SIQ/SIQ-JR by Reynolds might not be ideal. In fact, Reynolds’ factor analyses of the SIQ/SIQ-JR in the corresponding professional manual were exploratory, rather than confirmatory, since the questionnaires were not theoretically designed with distinct factors. Thus, a cautious claim was suggested by Reynolds that “use of these factors beyond simple descriptive purposes is not recommended at this time” [8].

EFA was thereafter deployed to provide insight of the underlying factor structure of both scales. A three-factor solution that accounted for 67.91% of the variance for the SIQ was identified (as shown in Table 1). This three-factor pattern, despite the difficulty to label each factor, was apparently not the same as Reynolds’ three-factor structure. The first factor might be interpreted as general wishes of suicide and response of others. The second factor could be referred to specific suicidal thoughts and plan. The third factor might be labeled as written signals of suicide and morbid ideation. EFA of the SIQ-JR yielded a two-factor solution that accounted for 67.76% of the variance (as shown in Table 2). A majority of 11 items fell into the first factor. These items indicated general wishes of suicidal ideation and plans, which could be roughly seen as a combination of the first and second factors in Reynolds’ three-factor classification. The other factor included the rest 4 items: item 8 (writing will), item 7 (writing suicide note), item 5 (thought of people dying), and item 9 (telling others), which might be interpreted as morbid ideation and written signals of suicide.

**Table 1:** **Rotated (Varimax) SIQ item factor loadings and communalities (h**^**2**^**)**

**Item description**	**Factor**			**h**^**2**^
	**I**	**II**	**III**	
28. If could kill self	0.80			0.76
29. If things didn’t improve	0.76			0.75
25. Others realize worth	0.74			0.71
14. Would solve problems	0.73	0.41		0.75
13. How easy it would be	0.70			0.68
23. Life too rotten	0.70	0.41		0.69
16. Wished had nerve	0.68			0.68
27. Thought of hurting self	0.64			0.67
26. No one cared if alive	0.60			0.56
10. Others happier if gone	0.60	0.58		0.75
24. Only way to be noticed	0.60			0.65
17. Wished never been born	0.58	0.44		0.60
30. Right to kill self	0.55	0.53		0.64
21. Having a bad accident	0.47			0.52
2. Thoughts of killing self		0.74		0.78
1. Better if not alive		0.70		0.62
12. Wished were dead	0.52	0.70		0.80
3. Thoughts of method	0.40	0.70		0.79
18. Would if had chance	0.59	0.64		0.82
4. Thought of time	0.44	0.63	0.40	0.75
15. Others better off	0.57	0.63		0.74
9. Telling others		0.61	0.47	0.62
22. Life not worth living	0.53	0.55		0.64
5. Thought of people dying			0.76	0.64
8. Writing will			0.74	0.67
7. Writing suicide note		0.52	0.64	0.74
11. How others would feel	0.53		0.55	0.67
20. Thought, but would not			0.54	0.55
19. Ways people kill themselves	0.49		0.52	0.56
6. Thought of death		0.49	0.50	0.60
Eigenvalue	17.95	1.35	1.07	
Variance (%)	59.84	4.51	3.57	∑ = 67.91

*Note*. Absolute values less than 0.40 were suppressed. All item descriptions are pphrased from actual scale.

**Table 2:** **Rotated (Varimax) SIQ-JR item factor loadings and communalities (h**^**2**^**)**

**Item description**	**Factor**		**h**^**2**^
	**I**	**II**	
11. Wished were dead	0.86		0.82
13. Others happier if gone	0.81		0.75
12. Would solve problems	0.77		0.68
1. Better if not alive	0.76		0.62
2. Thoughts of killing self	0.75	0.41	0.73
3. Thoughts of method	0.74	0.46	0.76
14. Wished never been born	0.74		0.62
4. Thought of time	0.69	0.52	0.74
15. No one cared if alive	0.66		0.55
6. Thought of death	0.63	0.46	0.61
10. How others would feel	0.62	0.50	0.63
8. Writing will		0.85	0.76
7. Writing suicide note	0.42	0.77	0.78
5. Thought of people dying		0.70	0.54
9. Telling others	0.44	0.64	0.60
Eigenvalue	9.11	1.05	
Variance (%)	60.75	7.01	∑ = 67.76

*Note*. Absolute values less than 0.40 were suppressed. All item descriptions are paraphrased from actual scale.

Different and uncertain patterns of factor structures for the SIQ/SIQ-JR were indicated by the comparison of the above results from both confirmatory and exploratory factor analyses with Reynolds’ solutions in the professional manual. Moreover, it is noticed that some other validation studies of the SIQ also yielded different solutions of factor structures. For example, Pinto et al. [10] supported a one-factor structure based on observation in a clinical sample that the first factor was sufficiently explanatory, which rendered additional factors extraneous. Another example was a study about academically gifted adolescents by Cassady and Cross [11], in which a four-factor structure was proposed. With regard to these inconsistent findings across studies, it is possible that there is lack of stable factorial structure for both scales. It is also possible that the factor construct varies in different populations. Given this consideration, it seems not wise insofar to generalize any specific patterns in explanation of both scales in future studies.

### SIQ-JR-4: a four-item short version of the SIQ-JR

A four-item short version of the SIQ-JR (SIQ-JR-4) was proposed by using the ranking of corrected item-total correlations, since this method can minimize the shared measurement error of item scores and total score [33]. This SIQ-JR-4 kept 4 items with the highest corrected item-total correlations (*r* > 0.8 for all 4 items in the sample) among all 15 items in the SIQ-JR, namely item 2 (I thought about killing self), item 3 (I thought about how I would kill myself), item 4 (I thought about when I would kill myself), and item 11 (I wished I were dead). These items have covered specific suicidal wishes and plans, as major aspects along the continuum of suicidal ideation [8].

### Concurrent validity

Spearman’s rank order correlations were calculated between the SIQ/SIQ-JR/SIQ-JR-4 and depression, hopelessness, and anxiety (results are displayed in Table 3). All correlations are significant at the *P* < 0.01 level. Moderate associations were found between the SIQ/SIQ-JR/SIQ-JR-4 and depression, hopelessness, and anxiety (*r* ranged 0.2-0.6). It is noticed that the SIQ-JR-4 was strongly correlated with the SIQ and SIQ-JR (*r* was 0.79 and 0.81 respectively). It is also noted that strength in correlations between the SIQ-JR-4 and depression, hopelessness, and anxiety was about the same as the correlations between the SIQ/SIQ-JR and the three psychological measurements.

**Table 3:** Spearman’s rank order correlations between SIQ, SIQ-JR, SIQ-JR-4 and psychological distress and well-being

	**SIQ**	**SIQ-JR**	**SIQ-JR-4**	**Depression**	**Hopelessness**	**Anxiety**
SIQ	1.00					
SIQ-JR	0.97	1.00				
SIQ-JR-4	0.79	0.81	1.00			
Depression	0.56	0.55	0.45	1.00		
Hopelessness	0.28	0.27	0.29	0.40	1.00	
Anxiety	0.46	0.45	0.33	0.59	0.26	1.00

*Note*. All correlations are significant at the *p* < 0.01 level. SIQ = Suicidal Ideation Questionnaire; SIQ-JR = Suicidal Ideation Questionnaire-Junior; SIQ-JR-4 = items 2, 3, 4, and 11 of the SIQ-JR.

**Figure 1:**
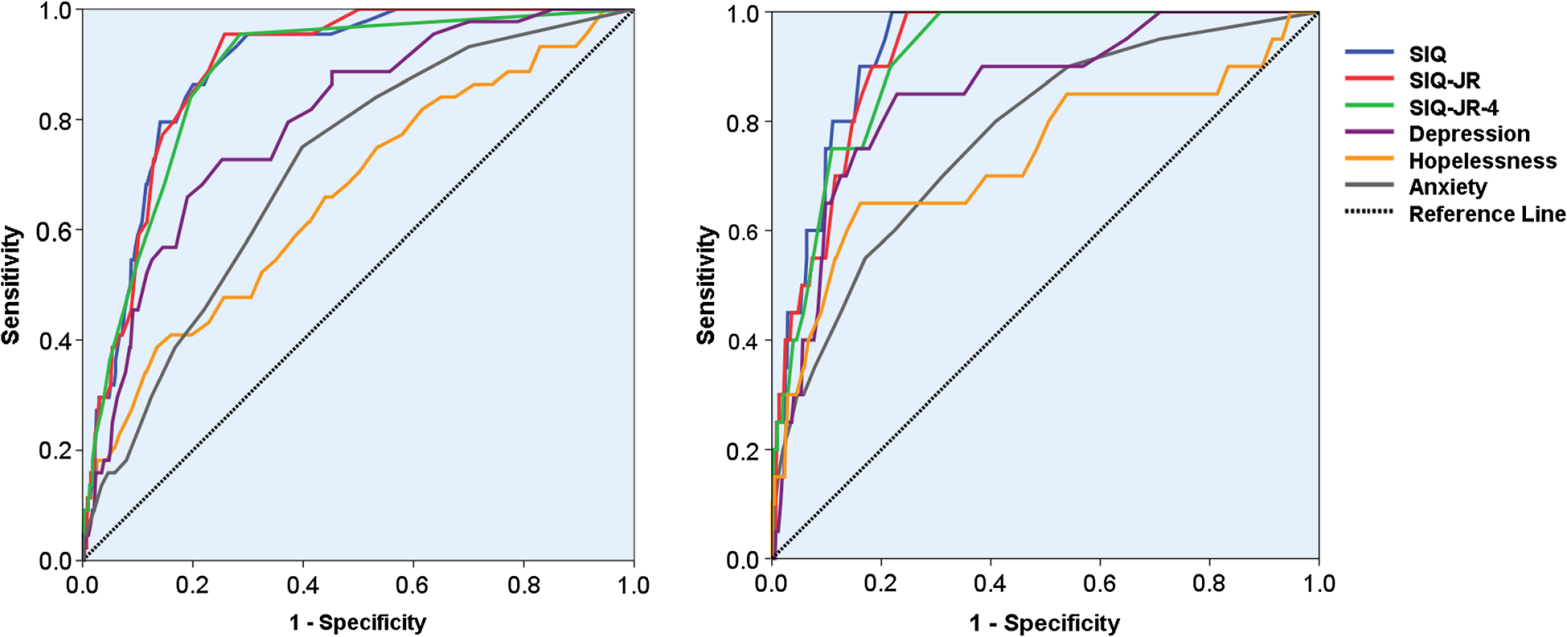
ROC curves for predicting suicidal ideation and suicide attempts in the past 12 months.

ROC analyses were performed using the SIQ, SIQ-JR, and SIQ-JR-4 to predict respondents with suicidal ideation and suicide attempts in the past 12 months in comparison with the predicting effects of depression, hopelessness, and anxiety (as shown in Table 4). The AUCs (area under curve) of the SIQ, SIQ-JR, and SIQ-JR-4 were larger than that of depression, hopelessness, and anxiety, which indicated better predicting effects of the three assessments of suicidal ideation. Correspondingly, the ROC curves of the SIQ, SIQ-JR, and SIQ-JR-4 were more away from the diagonal reference line than the other three measurements (as shown in Figure 1). For the screening of suicidal ideation in the past 12 months, no significant difference (*P* > 0.1) was detected among the AUCs of the SIQ, SIQ-JR, and SIQ-JR-4, yet they were all significantly (*P* < 0.05) larger than the AUCs of depression, hopelessness, and anxiety. Similarly, there was also no significant difference (*P* > 0.1) among the AUCs of the SIQ, SIQ-JR, and SIQ-JR-4 for screening suicide attempts in the past 12 months. The AUCs of the SIQ and SIQ-JR were significantly (*P* < 0.05) larger than those of depression, hopelessness, and anxiety. The AUC of the SIQ-JR-4 was also apparently (*P* < 0.05) larger than that of hopelessness and anxiety, though the difference of AUCs between the SIQ-JR-4 and depression did not reach the significant level of 0.05.

**Table 4:** ROC curves for predicting suicidal ideation and suicidal attempts in the last 12 months

**Measure**	**Area under curve**	**SE**^**a**^	**Asymptotic significance**^**b**^	**Asymptotic 95% CI**
*ROC curves for predicting suicidal ideation (44 suicidal thoughts out of 711)*				
SIQ	0.889	0.019	<0.001	0.852-0.926
SIQ-JR	0.891	0.018	<0.001	0.856-0.926
SIQ-JR-4	0.878	0.023	<0.001	0.832-0.923
Depression	0.795	0.033	<0.001	0.731-0.858
Hopelessness	0.661	0.045	<0.001	0.574-0.748
Anxiety	0.709	0.037	<0.001	0.636-0.782
*ROC curves for predicting suicide attempts (20 suicide attempts out of 711)*				
SIQ	0.928	0.016	<0.001	0.897-0.960
SIQ-JR	0.918	0.018	<0.001	0.882-0.953
SIQ-JR-4	0.912	0.020	<0.001	0.872-0.952
Depression	0.850	0.042	<0.001	0.767-0.932
Hopelessness	0.736	0.070	<0.001	0.599-0.873
Anxiety	0.772	0.053	<0.001	0.669-0.875

*Note.* SIQ = Suicidal Ideation Questionnaire; SIQ-JR = Suicidal Ideation Questionnaire-Junior; SIQ-JR-4 = items 2, 3, 4, and 11 of the SIQ-JR.

a. Under the nonpmetric assumption.

b. Null hypothesis: true area = 0.5.

Sensitivity and specificity analysis further confirmed that the four-item short version performed as good as the SIQ and SIQ-JR in screening suicidal ideation and suicide attempts (as shown in Table 5). By using cutoff score of 1, the SIQ-JR-4 successfully screened 42 out of 44 respondents who had suicidal ideation in the past 12 months (sensitivity = 0.95 and specificity = 0.71), and identified all 20 respondents who had suicide attempts in the past 12 months (sensitivity = 1 and specificity = 0.69). As a comparison, under cutoff score of 13, the SIQ’s results were “sensitivity = 0.86 and specificity = 0.80” for indicating past-12-month suicidal ideation, and “sensitivity = 1 and specificity = 0.78” for past-12-month suicide attempts; and under cutoff score of 6, the SIQ-JR’s results were “sensitivity = 0.95 and specificity = 0.74” for indicating past-12-month suicidal ideation, and “sensitivity = 1 and specificity = 0.72” for past-12-month suicide attempts. It is noticed that the positive predictive values (PPVs) of the three versions of SIQ were quite low. This was because of the low prevalence of suicidal ideation among the population sample. However, in consideration of the incremental validity, that is, the difference between the PPV and the base rate, all of the three scales performed quite satisfactory [41]. For example, for the SIQ-JR-4, which had the lowest PPVs among the three, its incremental validity for predicting suicidal ideation (18.0%-6.2% = 11.8%) and suicide attempts (8.6%-2.8% = 5.8%) were about twice as corresponding base rates.

**Table 5:** Sensitivity and specificity analysis of the SIQ, SIQ-JR, and SIQ-JR-4

	**SIQ (cutoff = 13)**^**a**^	**SIQ-JR (cutoff = 6)**^**a, b**^	**SIQ-JR-4 (cutoff = 1)**^**a**^
*Predicting suicidal ideation in the past 12 months (44 out of 711, prevalence rate = 6.2%)*			
Sensitivity	86.4%	95.5%	95.5%
Specificity	79.9%	74.2%	71.4%
Positive predictive value	22.1%	19.6%	18.0%
*Predicting suicide attempts in the past 12 months (20 out of 711, prevalence rate = 2.8%)*			
Sensitivity	100.0%	100.0%	100.0%
Specificity	78.0%	71.9%	69.2%
Positive predictive value	11.6%	9.3%	8.6%

*Note.* SIQ = Suicidal Ideation Questionnaire; SIQ-JR = Suicidal Ideation Questionnaire-Junior; SIQ-JR-4 = four-item short version of SIQ-JR (SIQ-JR items 2, 3, 4, and 11). a. Cutoff value was set at the value that maximized the Youden index (defined as “sensitivity + specificity-1”). b. Though in predicting suicide attempts in the past 12 months, cutoff of 7 had higher Youden index (0.75) than cutoff of 6 (Youden index = 0.72), cutoff for the SIQ-JR was decided at 6 for the purpose of consistent result in predicting suicidal ideation in the past 12 months.

In contrast, the rest three measurements (depression, hopelessness, and anxiety) performed less well, among which depression had the best predicting effects (under cutoff score of 13, “sensitivity = 0.73 and specificity = 0.75” for screening suicidal ideation, and “sensitivity = 0.85 and specificity = 0.73” for screening suicide attempts). These findings are very meaningful. They supported that the predicting and discriminative capacity of the SIQ-JR-4 was reasonably acceptable and hence the recommendation that it be a substitute of the SIQ/SIQ-JR for future quick assessment of suicidal ideation in young adolescents.

## Discussion

This study presents the psychometric properties of the Chinese version of the SIQ and SIQ-JR in a Hong Kong community sample of adolescents. The findings supported that the Chinese SIQ and SIQ-JR have excellent internal reliability and concurrent validity. However, the potential factor structure of both measurements remained unclear. The factor structure proposed by the original author is not recommended for future direct application before further validation. The results also suggested that the four-item short version of the SIQ-JR is a useful alternative to the SIQ and SIQ-JR. It demonstrated good concurrent validity in identifying respondents with suicidal ideation (sensitivity = 0.95 and specificity = 0.71) and suicide attempts (sensitivity = 1 and specificity = 0.69) in the past 12 months. Efforts on shortening of psycho-social measurement scales should be recommended so as to reduce burden on respondents and to increase response rate [23].

There are several limitations that should be carefully acknowledged. First, at least two concerns were raised due to the fact that the data employed for this study were a combined sample from two surveys. One issue is that the response rate of both surveys (61% and 62% respectively) was not very high. Yet, in consideration of the sensitive questions used, such response level was acceptable [16,42]. Another issue is that there were slight differences in sampling methods for the two surveys. For one sample source of our data, though a septe list of households from the Frame of Quarters was prepared, all adolescents aged between 15 and 19 years in these households were invited to be interviewed. This might lead to correlations in measurements among participants from the same household, as some previous studies had reported that there might be concordance for suicidal ideation between adolescent siblings in the same household [16,43]. Out of all 711 cases in the analysis, 140 (19.7%) were from the same household. Given the proportion of such cases was not very high, their influence of the findings in this study was considered in a low to modest level. Second, suicidality questions were self-reported in the household survey. Since there was lack of structured clinical diagnosis, the findings were subject to human response and memory bias. Third, the sample size of 711 for the analysis was not large. Therefore, statistical power might not be sufficient enough to detect some possible differences in measurements. For example, in predicting suicide attempts in the past 12 months, although the point estimates of AUCs for the SIQ-JR and SIQ-JR-4 were larger than that of depression, tests of difference between AUCs of these three measurements were not significant. Fourth, the survey did not actually administer the two short forms of the SIQ, namely the SIQ-JR and SIQ-JR-4. Instead, the full version of the SIQ was administered and the responses to the full 30-item questionnaire were used to obtain the scores of the two short versions. Future validation studies using randomized design to assign both full and short forms of the SIQ to participants will be useful.

Despite these limitations, there are many advantages for this study, including the community-based representative sample and a number of variables specifically designed to study suicidal ideation (e.g., depression, hopelessness and anxiety). A validated scale is highly needed for evaluating adolescent suicidality in Chinese-spoken societies. Additionally, this study provides a short form questionnaire as a convenient valuable tool for early screening of suicidal adolescents.

## Abbreviations

SIQ: Reynolds’ Suicidal Ideation Questionnaire; SIQ-JR: Reynolds’ Suicidal Ideation Questionnaire-Junior; SIQ-JR-4: Items 2, 3, 4, and 11 of the SIQ-JR; CES-D: Center for epidemiologic studies depression scale; BHS: Beck’s hopelessness scale; DASS: Depression anxiety stress scale; SPSS: Statistical package for the social sciences; AMOS: Analysis of moment structures; EFA: Exploratory factor analysis; CFA: Confirmatory factor analysis; ROC: Receiver operating characteristics; AUC: Area under ROC curve; RMSEA: Root-mean-square error of approximation; GFI: Goodness-of-fit index; CFI: Comptive fit index; PPV: Positive predictive value

## Competing interests

The authors declare that they have no competing interests.

## Authors’ contributions

YZ, PSFY and KF together designed the study. YZ analyzed the data and wrote the draft of the paper. All three authors have given final approval of this version to be published.
